# Analysis of smoke cessation rate and profile of former smokers living in Belo Horizonte and Metropolitan Region

**DOI:** 10.1590/S1679-45082014GS2899

**Published:** 2014

**Authors:** Luana Maria Oliveira Claudino, Mery Natali Silva Abreu

**Affiliations:** 1 Universidade Federal de Minas Gerais, Belo Horizonte, MG, Brazil

**Keywords:** Smoking habit cessation, Smoking habit

## Abstract

**Objective:**

To estimate the smoking cessation rate and to identify possible associated factors.

**Methods:**

Individuals from the Metropolitan Region of Belo Horizonte (MG) aged 15 years or more who responded to the smoking supplement of the National Household Sample Survey were selected (n=1,297). An estimate was made of the tobacco-use cessation rate relative to the 95% confidence interval. To verify factors associated with smoking cessation, Pearson's χ^2^ test or Student's t test were used.

**Results:**

The general smoking cessation rate was 56.7% (95% confidence interval: 52.3-61.1), with 57.7% among men and 55.5% among women. The associated factors were higher age and income. Among the 19 individuals who had stopped smoking for less than 12 months, 52.6% had been seen by a physician or healthcare professional in the previous 12 months, and 60% of them were oriented to stop smoking, but only 1 (16.7%) had been oriented at a unit of the public national Unified Healthcare System.

**Conclusion:**

Despite high rates of smoking cessation, the methods made available by the Unified Healthcare System did not show good results. It is necessary to enhance the healthcare programs that aim to reduce the proportion of smokers in the population. Such measures can have a positive long-term influence in droping mortality and morbidity rates associated with smoking and the costs for healthcare services.

## RESUMO

**Objetivo:**

Estimar o índice de cessação do tabagismo e identificar possíveis fatores associados.

**Métodos:**

Foram considerados os indivíduos da Região Metropolitana de Belo Horizonte (MG) com 15 anos de idade ou mais, que responderam ao questionário da Pesquisa Nacional por Amostra de Domicílios no suplemento sobre tabagismo (n=1.297). Foi realizada estimativa do índice de cessação do tabaco com respectivo intervalo de confiança de 95%. Para verificar fatores associados à cessação do tabagismo, realizou-se o teste χ^2^ de Pearson ou *t* Student.

**Resultados:**

O índice geral de cessação do tabaco foi de 56,7% (intervalo de confiança de 95%: 52,3-61,1), sendo de 57,7% entre os homens e de 55,5% entre as mulheres. Os fatores associados à cessação foram maior idade e renda. Entre os 19 indivíduos que pararam de fumar há menos de 12 meses, 52,6% foram atendidos por médico ou profissional de saúde nos últimos 12 meses, dos quais 60% foram aconselhados a parar de fumar, mas apenas 1 (16,7%) foi aconselhado no Sistema Único de Saúde.

**Conclusão:**

Apesar dos altos índices de cessação do tabagismo, os métodos disponibilizados pelo Sistema Único de Saúde não apresentaram bons resultados. Faz-se necessário um aumento dos programas de saúde que têm como objetivo a redução do percentual de fumantes na população. Tais medidas podem repercutir positivamente a longo prazo, na redução das taxas de mortalidade e morbidade associadas ao tabagismo e nos custos para os serviços de saúde.

## INTRODUCTION

Cigarettes are among the most often used drug throughout the world, and are responsible for approximately 50% of five million deaths registered in the year 2000 in developing countries.^(1)^ The habit of smoking is associated with the causes of respiratory and cardiac diseases, cerebrovascular accidents (stroke), peptic ulcer, upper respiratory tract cancer,^(2)^ and superficial bladder cancer.^(3)^

According to data collected in Brazil the capital cities where the adult population smokes more are Porto Alegre (RS) with 23%, Curitiba (PR) with 20%, and São Paulo (SP), with 19%. On the other hand, the Northeast regions show the lowest rates of smokers per capital city: Maceió (AL) had a prevalence of 8% and João Pessoa (PB), Aracajú (SE), and Salvador (BA), of 9% of smokers.^(4)^

In 2003, Brazil signed, along with 192 countries, the Framework Convention on Tobacco Control (FCTC) that proposes international multilateral collaboration obligations and intersectoral actions to review the situation of smoking in the participating countries. This convention determined that the national agenda include the national policy to fight tobacco use. In this way, the country launched its systematic fight, promotion of cessation, and systematic tobacco-use control. This resulted in public healthcare policies, awareness actions for the population, financing actions for smoking control in the Unified Healthcare System (SUS), and the control and supervision of tobacco products in the country.^(5)^

Recognizing the damaging power of the use of smoked tobacco and the high cost of treatment in the public health sector suggest strategies to lessen this situation. The most successful policies of tobacco control in the world point out that prior attempt is an important factor for maintenance of cessation.^(6,7)^ As to the quitting smoking, in 2007, Belo Horizonte (MG) had smoking cessation rates of 21.6% – similar to those of the adult population of the United States. (8) The smoking cessation rate is understood as “the percentage of former smokers among those who have already smoked at some time during their lives.”^(8)^

## OBJECTIVE

Considering what was exposed and using data from the National Household Sample Survey, of 2008, this study had the objective of estimating the smoking cessation rate, identifying the primary methods for smoking cessation, and the profile of former smokers among the residents of Belo Horizonte and its Metropolitan Region.

## METHODS

This is an observational cross-sectional study carried out with the data from the National Household Sample Survey (PNAD, acronym in Portuguese) in its Special Smoking Research (PETab, acronym in Portuguese).^(9)^

Since it deals with public domain data, this study was exempt from requiring approval from the Research Ethics Committee (CEP).

The study considered individuals living in Belo Horizonte and its Metropolitan Region (RMBH), aged 15 years or more. This sample was composed of 26 municipalities, 248 sectors, and 4,693 household units. The sample comprised 1,297 individuals who responded to chapter 27 of the PNAD questionnaire, entitled “Special Investigation of Smoking in Residents aged 15 years or More” (*i.e*., born up to Sep 27, 1993).^(10)^

### Instruments and procedures for data collection

The PETab was a household investigation with a sampling plan similar to that of PNAD. The sampling plan of PNAD consisted of a probability sample of households with three stages of selection: primary units – municipalities; secondary units – census districts; and tertiary units – household units (*i.e*., private households). In the first stage, the units (municipalities) were classified into two categories: self-representative (for example: probability 1 of belonging to the sample) and non-self-representative. The municipalities of the second category went through a stratification process, and at each stratum, two cities were selected without reposition and with a probability proportional to the resident population obtained in the last demographic census (for example: the 2000 Demographic Census). In the second stage, the units (for example: census districts) were selected in each municipality of the sample, with a probability proportional and without reposition, using the number of existing household units at the moment of the 2000 Demographic Census as a measurement of size. In the third stage, with equiprobability, private households and housing units in collective households were selected for investigation of the characteristics of the residents and of housing. The PETab questionnaire was answered by the resident selected, which implied a modification in the standard methodology of PNAD that uses a proxy informant in order to obtain information on the residents who are not present at the time of the interviewer's visit to the house. Therefore, PETab was applied to a subsample of households of PNAD in all the sectors that belonged to the sample of this research.^(10)^

### Variables studied

The variable answer of the study was the cessation of tobacco use, and former smokers were considered as all those non-smokers who had already smoked at some time during their lifetime.^(8)^

As possible factors associated with smoking, sociodemographic and socioeconomic variables were analyzed, such as gender, age, *per capita* income, and level of schooling in number of years of study.

In order to identify some of the characteristics of former smokers as well as the methods used in stopping the smoking habit, a few variables were used, such as the unit of measure used to indicate the time since the individual quitted smoking (*i.e*., v2731); if there was any SUS visit in which the individual was advised to stop smoking (*i.e*., v2726); if in the last 12 months, as a procedure to stop smoking, the individual used nicotine replacement tehrapy (*i.e*., v7222); if over the last 12 months such a procedure to stop smoking utilized teas, herbs, or medicinal plants (*i.e*., v7225); if over the last 12 months, as a procedure to quit smoking, telephone help resources were used (*i.e*., v7226); number of months without smoking during the last time the individual tried to stop smoking (*i.e*., v7244); if currently he/she smokes any kind of tobacco (*i.e*., v2701); if in the past he/she smoked any tobacco product (*i.e*., v2703).

### Statistical analysis

Initially, an estimate was made of the tobacco cessation rate in the population studied with respect to a 95% confidence interval (95%CI), besides a descriptive analysis of all variables studied.

In order to verify factors associated with smoking cessation, a univariate analysis was made, using Pearson's χ2 test for the categorical variables, or Student's t test for the continuous variables.

In the multivariate analysis, a Poisson regression model was constructed with robust variances. The model adjustment was made by using the backward criterion, with removal of the variables one by one, according to their significance; those that remained in the model had a value of p<0.05. The values of the prevalence ratio (PR) with a respective 95%CI were estimated.

Data were analyzed using Stata10^®^ software. All analyses took into consideration the weights imposed by the sample design and were used with a significance level of 5%.

## RESULTS

According to the results shown on table 1, the sample for the present study included 1,297 residents of RMBH, who answered PETab – PNAD 2008. Distribution by gender corresponded to 53.9% of women. The mean *per capita* income was R$ 920.30. The mean age was 41.8 years, and the level of schooling was 9.2 years of study.

**Table 1:** Descriptive statistics of the demographic and socioeconomic factors, Metropolitan Region of Belo Horizonte, 2008 (n=1,297)

Characteristics		mean (±DP)	Minimum	Maximum
Age (years)		41.8 (±17.0)	15	97
Schooling (years)		9.2 (±4.3)	1	17
Income *(per capita)*		R$ 920.30 (±1,782.2)	R$ 00.00	R$ 33,000.0
Gender (%)				
	Female	699 (53.9)		
	Male	598 (46.1)		

SD: standard deviation.

As per the results presented on table 2, the percentage of daily and current use of tobacco was 14.4%. Considering the total number of current smokers, one can affirm that the prevalence of smoking was 16.7%.

**Table 2:** Descriptive statistics associated with smoking, among individuals aged 15 years or more, in the Metropolitan Region of Belo Horizonte, 2008

Prevalence of smoking among individuals aged 15 years or more, Metropolitan Region of Belo Horizonte, 2008 (n=1,297)		n (%)
Currently smokes		
	Daily	187 (14.4)
	Less than daily smoking	29 (2.2)
	Does not smoke	1,081 (83.4)
Former smokers among individuals aged 15 years or more who reported not currently smoking, Metropolitan Region of Belo Horizonte 2008 (n=1,081)		n (%)
	Former smoker	283 (26.2)
	Never smoked	798 (73.8)
Former smokers among individuals aged 15 years or more who reported not being current smokers, Metropolitan Region of Belo Horizonte 2008 (n=1,081)		n (%)
	Does not currently smoke	283 (56.71)
	Current smoker	216 (43.29)
	Does not currently smoke	283 (56.71)

Prevalence de cessation: 95%CI: 52.3-61.1%.

Of the 1,081 individuals who reported not currently smoking, 798 (73.8%) never smoked. On the other hand, 283 were former smokers, with a total of 499 interviewees who had already smoked at some time during their lives. In this way, the tobacco cessation rate among individuals aged 15 years or more of the RMBH was 56.7% (95%CI: 52.3-61.1). As demonstrated on table 3, a significant difference was observed between the ages of smokers and former smokers (p<0.05). The mean age of former smokers was higher if compared to that of smokers. There was also a significant difference for the income of former smokers, which proved to be higher when compared to that of smokers. As to the level of schooling, there was no significant difference between the groups (p>0.05). The percentages of cessation of 57.7% among men and 55.5% among women were noted. However, this comparison was not significantly different (p>0.05).

**Table 3:** Univariate analysis of demographic and socioeconomic factors in current smokers and former smokers aged 15 years or more, Metropolitan Region of Belo Horizonte, 2008 (n=499)

		current smoker	Former smoker	p value
		Mean (±SD)	Mean (±SD)	
Age (years)		41.1 (±13.9)	49.9 (±16.1)	<0.001*
Income *(per capita)*		R$ 677.20 (±839.5)	R$ 1,245.30 (±2,401.7)	<0.001*
Schooling (years)		8.6 (4.3)	8.5 (4.6)	<0.950*
Gender (%)				
	Female	98 (44.6)	122 (55.5)	<0.254**
	Male	118 (42.3)	161 (57.7)	

SD: standard deviation.

* Student's t test;

** Pearson's χ^2^ test.

Also presented was the time of cessation of the former smokers, with the documentation that most (93.3%) had quitted smoking for over one year. Analysis of the methods used for smoking cessation was made only for those who stopped smoking less than 12 months before (n=19), since the question of that item was directed only to former smokers who had stopped less than one year before. Among these, 52.6% (n=10) had been seen by a physician or healthcare professional in the previous 12 months, 60% (n=6) of them instructed to stop smoking, but only 16.7% (n=1) had been instructed at SUS.

A few methods were identified that had been used by the individuals to stop smoking over the previous 12 months, among them: nicotine replacement with patches, lozenges, sprays, inhalers, or chewing gum; other medications with medical prescriptions; homeopathy and acupuncture; teas, herbs, or medicinal plants; telephone help services; exchange for another tobacco product that does not produce smoke, besides any other not mentioned in the questionnaire.

The only method reported among the 19 former smokers who had stopped for less than one year was the telephone help service, reported by one individual (5.3%). Four individuals (21.0%) reported other methods not mentioned in the questionnaire. None had been instructed by SUS.

Also in the multivariate analysis (Table 4), there was a greater probability of the individual being a former smoker among those with older (PR=1.013) and greater incomes (PR=1.040). The increase of one unit in age increased by 1.3% the probability of being a former smoker and the increase of R$1,000.00 in income increased this probability by 4%.

**Table 4:** Multivariate analysis by means of Poisson's regression model of the demographic and socioeconomic factors associated with the condition of former smokers among residents aged 15 years or older in the Metropolitan Region of Belo Horizonte, in 2008 (n=499)

	PR	95%CI	p value
Age (1 year increase)	1.013	1.001-1.018	<0.001
Income (R$ 1,000.00 increase)	1.040	1.024-1.057	<0.001

PR: prevalence ratio; 95% CI: 95% confidence interval.

## DISCUSSION

The cessation index rate among individuals who reside in the RMBH was high, and cessation showed a significant association with higher age and income level. Additionally, the only method used to stop smoking reported by the former smokers who had quitted less than 12 month before was the telephone help service.

It may be observed that the cessation rate in the RMBH was high relative to the values found in other countries, such as Spain, where the population aged over 15 years showed a cessation rate of 32.4% in Catalonia and 40.1% in Barcelona. In the United States, the rate was 50.3% in the adult population and, in Brazil, there is a variation between 44% and 58.3% in the capital cities of the research made by the Household Survey on Reported Risk Behaviors and Morbidity for Noncommunicable Diseases and Illnesses: Brazil, 15 capital cities, and the Federal District.^(8)^

Our study shows a cessation rate in the RMBH for men and women higher than the results presented by Giovino et al.^(11)^ in a study that evaluated adults older than 15 years, in 16 countries including Brazil. In the said study, the rate of former male smokers in Brazil was 46.4% (95%CI: 44.9%-47.8%), and 47.7% (95%CI: 46.0%-49.4%) for women. This index places Brazil as the third country with the highest rate of former smokers, ranking behind the United Kingdom (men with 57.1%, and women with 51.4%) and the United States (men with 48.7%,and women with 50.5%).

In the analysis of factors related to smoking cessation, a higher age among former smokers was noted. According to Peixoto et al.,^(8)^ the cessation rate may be influenced by age due to three factors: (1) by being a cumulative measure, the probability of older individuals reporting interruption of the smoking habit is greater among those who are younger; (2) the habit of smoking reduces with age as a consequence of health problems and/or due to concerns about health; and (3) survival bias, due to greater survival of former smokers when compared to individuals who continue to smoke.

Another factor that showed a significant association with smoking cessation was income. Otero et al.^(12)^ pointed out the importance of discussing the involvement of SUS among people with low incomes, since this is a population that has shown the lowest rate of smoking less.

In this study, no association was found between smoking cessation and the variables of gender and level of schooling. However, Peixoto et al.^(8)^ reported that such an association is found in developed countries, where cessation is greater among older individuals, with a higher level of education and income, and in general, it is believed that people with less education find it more difficult to stop smoking due to low motivation and lack of resources.

Counseling for quitting smoking offered by SUS professionals, according to the present study, did not show good results, since none of the individuals reported having been instructed to stop smoking by SUS professionals, nor had they used methods for smoking cessation offered by the public healthcare system. Despite the results found in our study, Otero et al.^(12)^ highlighted that an intensive approach in treating a smoker, along with the use of patches, has a positive correlation with smoking cessation 12 months after the intervention.

With this perspective, Carvalho^(13)^ reported that tobacco control strategies should foresee the identification of smokers to reach those who desire to stop smoking, as well as the fact that cessation therapies adopted by the government, at any level of power, cannot prioritize “this or that therapy” since their efficacy, acceptability, and cost/effectiveness ratio may vary. Strengthening public healthcare management is required to overcome the difficulties (decentralization of the system, political and administrative spheres, and regional heterogeneity) and inequalities (social, cultural, and economic) found in the Brazilian scenario.

We should further point out the importance of the role of public agencies regarding health promotion strategies, especially in the low income population – strategies that seem to not be producing the efficacy necessary, since, as has been previously mentioned, the methods for smoking cessation offered by SUS have not been reported by the former smokers analyzed in this study.

As emphasized by Abreu et al.,^(14)^ healthcare managers must propose policies that fight smoking, with actions that go beyond the individual level since it is necessary to develop interventions geared towards the community, school, and family, which should be carried out at the level of Primary Health Care.

According to the Final Report, Burden of Tobacco-Related Diseases in Brazil*,* coordinated by the Alliance for Smoking Control*,* ^(5)^ the costs last year, both for SUS and for supplementary health services, were R$ 21 billion with the evils caused by cigarettes, especially with heart diseases, chronic obstructive pulmonary diseases, lung cancer, and stroke, which together correspond to 83% of expenses. In this way, managers should increase investment in prevention campaigns against smoking, which could further reduce expenditures with problems related to smoking.

The study has limitations, since the outcome was evaluated by means of answers to a questionnaire, and therefore, the prevalence of smoking may be underestimated since only selected residents aged 15 years or older could answer the questionnaire, and there could be circumvention of information.

Since it is a cross-sectional study, aging could not be used as a causal criterion, since event and outcome are considered at the same time point.

Additionally, due to the fact that the questionnaire items were directed only towards methods used by former smokers with less than 12 months of cessation, in 93.29% of the interviewees it was not possible to apply methods made available by SUS. This precluded greater analysis and discussion on the subject.

## CONCLUSION

The present study identified a high rate of cessation in the adults aged 15 years or older, who reside in Belo Horizonte and Metropolitan Region in 2008. Nevertheless, the methods made available by the Unified Healthcare System for smoking cessationdid not show good results, taking into consideration the evaluation instrument used in data collected. With this, an increase in health programs with the objective of reducing the percentage of smokers in the population is needed. Such measures may have a positive long-term repercussion in the reduction of mortality and morbidity rates associated with smoking, and in costs for healthcare services.

## References

[1] World Health Organization (WHO) (2001). World Health Organization Press [Internet].

[2] World Health Organization (WHO) (2002). World Health Organization Press [Internet].

[3] Korkes F, Juliano CA, Bunduky MA, Costa AC, Castro MG (2010). Associação entre o consumo de tabaco e a progressão do câncer superficial da bexiga. einstein.

[4] Instituto Nacional de Câncer (INCA) (2012). INCA.

[5] Aliança de Controle do Tabagismo no Brasil (ACTBR) (2011). ACTBR.

[6] Figueiredo VC (2007). Um panorama do tabagismo em 16 capitais brasileiras e Distrito Federal: tendência e heterogeneidades.

[7] Hyland A, Borland R, Li Q, Yong HH, McNeill A, Fong GT (2006). Individual-level predictors of cessation behaviours among participants in the International Tobacco Control (ITC) Four Country Survey. Tob Control.

[8] Peixoto SV, Firmo JO, Lima-Costa MF (2007). Fatores associados ao índice de cessação do habito de fumar em duas populações adultas (Projeto Bambuí e Belo Horizonte). Cad Saúde Pública.

[9] Instituto Brasileiro de Geografia e Estatística (IBGE) (2008). Pesquisa Nacional por Amostra de Domicílios (PNAD) [Internet].

[10] Instituto Nacional de Câncer (INCA) (2011). INCA.

[11] Giovino GA, Mirza SA, Samet JM, Gupta PC, Jarvis MJ, Bhala N (2012). Tobacco use in 3 billion individuals from 16 countries: an analysis of nationally representative cross-sectional household surveys. Lancet.

[12] Otero UB, Perez Cde A, Szklo M, Esteves GA, dePinho MM, Szklo AS (2006). Ensaio clínico randomizado: efetividade da abordagem cognitivocomportamental e uso de adesivos transdérmicos de reposição de nicotina, na cessação de fumar, em adultos residentes no Município do Rio de Janeiro, Brasil. Cad Saúde Pública.

[13] Carvalho CR (2009). O Instituto Nacional de Câncer e o Controle do tabagismo: uma análise da gestão federal do tratamento do tabagismo no SUS.

[14] Abreu MN, Caiaffa WT (2011). Influência do entorno familiar e do grupo social no tabagismo entre jovens brasileiros de 15 a 24 anos. Rev Panam de Salud Publica.

